# Identification and Validation of Plasma Protein Biomarkers for Abdominal Aortic Aneurysm Using Integrated Proteomics

**DOI:** 10.3390/ijms27167312

**Published:** 2026-08-16

**Authors:** Huibo Ma, Jianhang Gao, Yihang Cai, Zongyou Xie, Lianglin Wu, Wenxuan Xiang, Xiaohong Song, Bintao Qiu, Fangda Li, Jianqiang Wu, Yuehong Zheng

**Affiliations:** 1 Department of Vascular Surgery, Peking Union Medical College Hospital, Chinese Academy of Medical Sciences and Peking Union Medical College, Beijing 100730, China; mhb_doctor@163.com (H.M.); billgao99@icloud.com (J.G.); b2025001046@student.pumc.edu.cn (Y.C.); xiezy_we95@163.com (Z.X.); pumc_wulianglin@student.pumc.edu.cn (L.W.); xiangwx@student.pumc.edu.cn (W.X.); xhsongimc@163.com (X.S.); lifangda@pumch.cn (F.L.); 2 Center for Biomarker Discovery and Validation, National Infrastructures for Translational Medicine, Institute of Clinical Medicine, Peking Union Medical College Hospital, Chinese Academy of Medical Sciences and Peking Union Medical College, Beijing 100730, China; qiubintao@pumch.cn; 3 State Key Laboratory of Complex Severe and Rare Diseases, Peking Union Medical College Hospital, Chinese Academy of Medical Sciences and Peking Union Medical College, Beijing 100730, China

**Keywords:** abdominal aortic aneurysm, proteomics, biomarkers, pathogenesis, therapeutic targets

## Abstract

Abdominal aortic aneurysm (AAA) is a progressive and often asymptomatic vascular disease associated with high mortality after rupture, but reliable circulating biomarkers for noninvasive detection remain limited. We aimed to identify and validate plasma protein biomarkers for AAA using an integrated proteomics-based approach. Plasma samples from 22 patients with AAA and 22 healthy controls were analyzed through data-independent acquisition (DIA) mass spectrometry. Differentially expressed proteins were subjected to bioinformatic analyses, including Gene Ontology enrichment, Kyoto Encyclopedia of Genes and Genomes pathway analysis, protein–protein interaction, and weighted gene coexpression network analyses. Candidate biomarkers were selected on the basis of differential abundance, diagnostic performance, and biological relevance and subsequently validated by enzyme-linked immunosorbent assay in an independent cohort comprising 93 patients with AAA and 83 non-AAA controls. DIA proteomics identified 111 differentially abundant proteins, revealing enrichment of pathways related to mitochondrial respiration, oxidative stress, inflammation, extracellular matrix remodeling, and proteostasis. Among the candidates, plasma CHRDL1 levels were significantly reduced, whereas OGN and CCL18 levels were significantly elevated in patients with AAA; these findings were consistently confirmed in the validation cohort. A combined three-protein model demonstrated strong diagnostic performance, with an area under the receiver operating characteristic curve of 0.890. These findings identify CHRDL1, OGN, and CCL18 as promising plasma biomarkers for AAA detection and further highlight mitochondrial dysfunction, chronic inflammation, ECM remodeling, and dysregulated proteostasis as key molecular features of AAA.

## 1. Introduction

Abdominal aortic aneurysm (AAA) is a common degenerative vascular disease characterized by permanent, localized dilation of the abdominal aorta, usually defined as an abdominal aortic diameter of ≥30 mm on imaging [1,2]. AAA usually develops silently and remains asymptomatic until late stages. However, once the aneurysm ruptures, the mortality rate is extremely high [3,4]. As the population ages, the prevalence of AAA is increasing, posing a growing public health burden [5]. Currently, the diagnosis and surveillance of AAA depend mainly on imaging modalities, including abdominal ultrasound and computed tomography angiography (CTA). Although these techniques are clinically effective, they have several limitations. Ultrasound is operator-dependent, whereas CTA is relatively costly and involves radiation exposure and potential contrast-associated complications [6,7]. These limitations may restrict their broader application in population-based screening and repeated monitoring. Therefore, identifying convenient, noninvasive, and reliable circulating biomarkers may serve as a complementary approach for AAA detection while improving our understanding of disease-related molecular alterations.

Studies have reported several circulating molecules associated with AAA, including matrix metalloproteinases, D-dimers, inflammatory cytokines, and other proteins involved in vascular remodeling [8,9,10]. These biomarkers partially reflect pathological processes such as extracellular matrix (ECM) degradation, vascular inflammation, oxidative stress, and vascular smooth muscle cell dysfunction. Nevertheless, the pathogenesis of AAA is highly complex and involves coordinated interactions among multiple cellular and molecular pathways [11,12]. Consequently, individual candidate biomarkers identified from hypothesis-driven studies often have limited diagnostic sensitivity or specificity, which restricts their clinical translation. These observations highlight the need for systematic and unbiased approaches to explore the plasma protein alterations associated with AAA.

Recent advances in omics technologies have provided powerful tools for comprehensive biomarker discovery. Genome-wide association studies have identified numerous genetic loci associated with AAA susceptibility, suggesting a complex molecular basis for this disease [13]. In parallel, high-throughput proteomic technologies have enabled large-scale profiling of circulating proteins and have provided new opportunities to identify disease-associated biomarkers directly from plasma or serum [14]. Among these approaches, data-independent acquisition (DIA) mass spectrometry has emerged as a robust and reproducible technique for quantitative proteomic analysis, allowing broad coverage of the plasma proteome and systematic comparisons between disease and control groups [15]. However, proteomic discovery alone is insufficient for biomarker development, and candidate proteins require further validation using targeted quantitative methods. Enzyme-linked immunosorbent assay (ELISA) remains among the most widely used approaches for experimental validation because of its accessibility, reproducibility, and potential clinical applicability [16,17]. Although several proteomic studies have explored circulating proteins associated with AAA, many reported candidates have not been independently validated in clinical plasma samples [14,18,19]. Moreover, studies integrating unbiased proteomic discovery with subsequent experimental validation remain limited. Therefore, further investigations are needed to identify reproducible plasma biomarkers that distinguish patients with AAA from healthy individuals and to provide biological insights into disease-associated protein alterations.

In the present study, we performed a plasma proteomic analysis of patients with AAA and healthy volunteers through DIA mass spectrometry. Differentially expressed proteins (DEPs) were systematically identified, and bioinformatics analyses were then performed to characterize the biological processes and molecular pathways associated with AAA. Candidate proteins with potential diagnostic value were then selected and validated in an independent set of plasma samples using ELISA. Furthermore, the potential functional roles of validated AAA biomarkers were explored using public single-cell RNA-sequencing data for AAA and in silico knockout analysis. Through this discovery-to-validation workflow, we aimed to identify novel plasma biomarkers for AAA and provide additional insight into the molecular mechanisms underlying AAA progression.

## 2. Results

### 2.1. Participant Characteristics in the Discovery and Validation Cohorts

**Figure 1:**
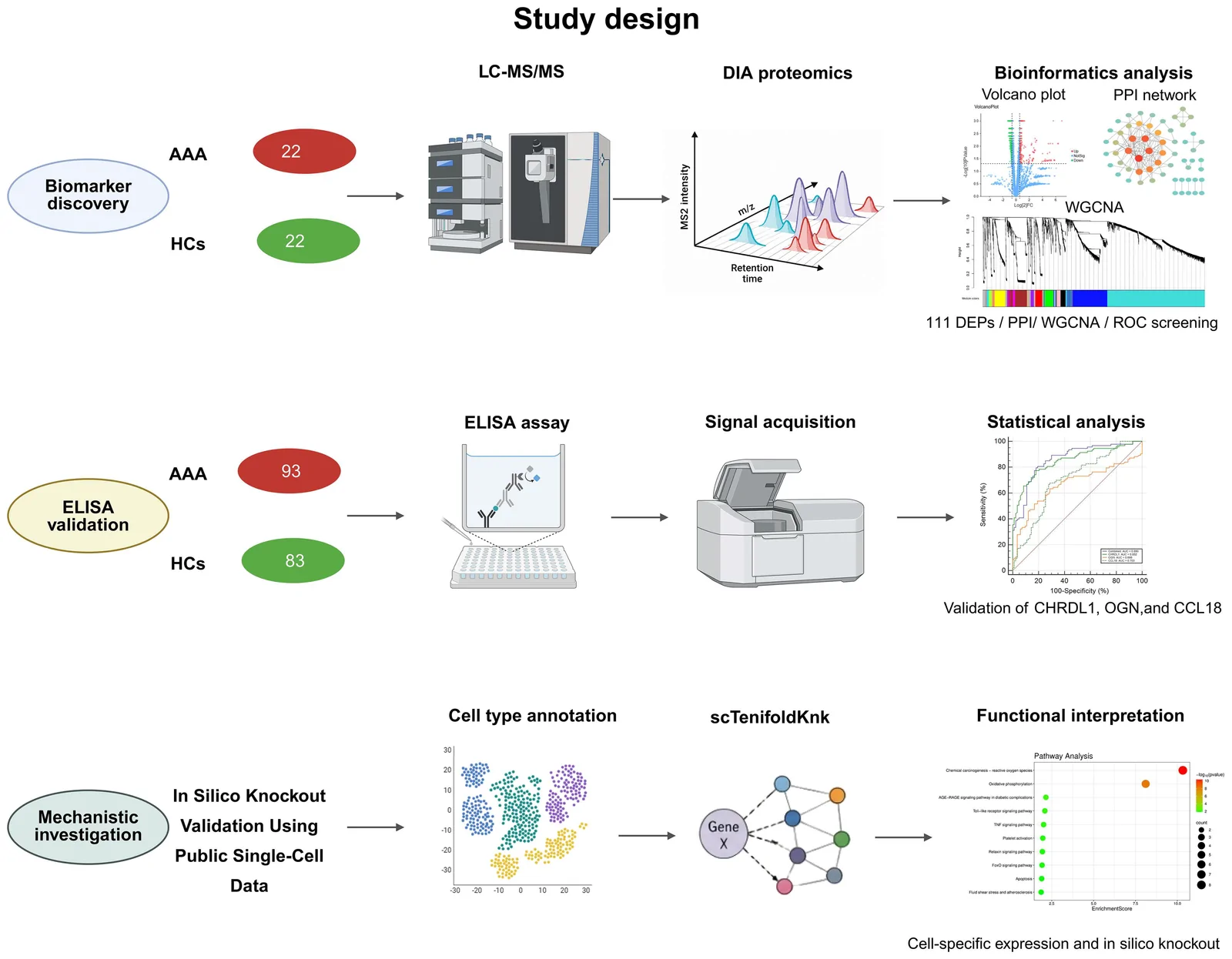
Study design and workflow in this study.

A schematic diagram of the study design is presented in Figure 1. A total of 115 patients with AAA and 105 non-AAA controls were included in this study. Among them, 22 patients with AAA and 22 HCs were included in the proteomic discovery cohort, while the remaining 93 patients with AAA and 83 controls were included in the ELISA validation cohort. All participants in the discovery and validation cohorts were recruited from the outpatient and inpatient services of the Department of Vascular Surgery of Peking Union Medical College Hospital (PUMCH).

The baseline characteristics of the participants in both cohorts are summarized in Table 1. In the proteomic discovery cohort, no significant differences were observed between the AAA patients and HCs with respect to age, sex, smoking status, or hypertension, diabetes mellitus, chronic kidney disease, or chronic obstructive pulmonary disease status. In the ELISA validation cohort, age and sex were comparable between the AAA patients and non-AAA controls. Compared with the controls, patients with AAA had a significantly greater prevalence of smoking history (57.0% vs. 38.6%, *p* = 0.015). No significant differences in hypertension, diabetes mellitus, chronic kidney disease, or chronic obstructive pulmonary disease status were detected between the two groups.

### 2.2. Distinct Plasma Proteomic Profiles Were Identified in Patients with AAA

**Figure 2:**
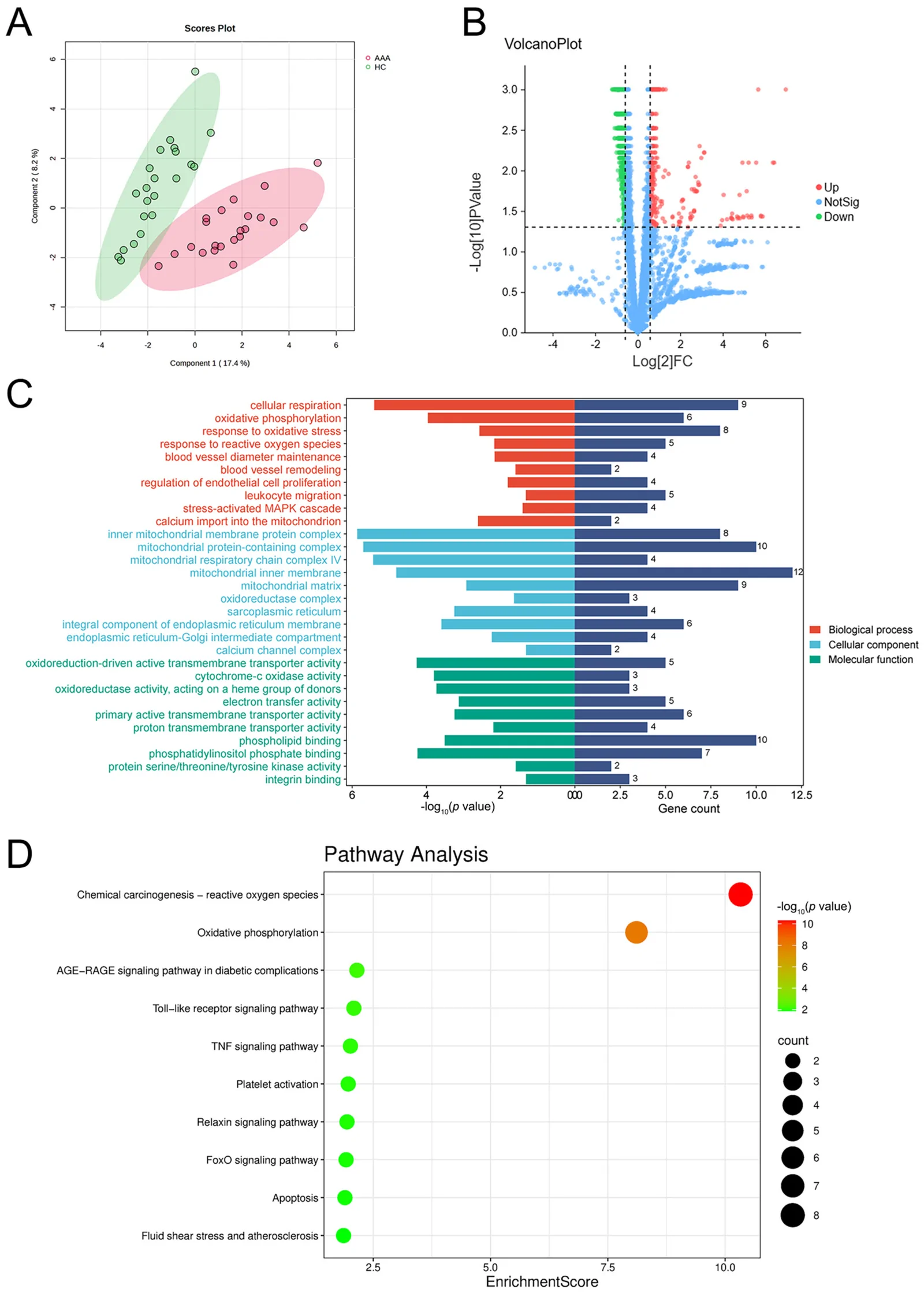
DIA-based plasma proteomic analysis and enrichment analysis of DEPs in AAA. (**A**) Partial least squares discriminant analysis (PLS-DA) score plot of plasma proteomic profiles from abdominal aortic aneurysm (AAA) patients and healthy controls (HCs). (**B**) Volcano plot of differentially expressed proteins (DEPs) between AAA patients and HCs. Red dots indicate upregulated proteins, green dots indicate downregulated proteins, and blue dots indicate proteins that exhibited no significant differential expression. (**C**) Gene Ontology (GO) enrichment analysis of the DEPs, including the biological process, cellular component, and molecular function categories. (**D**) Kyoto Encyclopedia of Genes and Genomes (KEGG) pathway enrichment analysis of the DEPs. The bubble size represents the number of enriched proteins, and the color indicates the significance level expressed as −log_10_(*p* value).

In the biomarker discovery cohort, DIA-based quantitative proteomics analysis identified 4106 plasma proteins with an FDR of less than 1%. Partial least squares discriminant analysis (PLS-DA) revealed significantly different protein expression patterns between AAA patients and HCs (Figure 2A).

Using the predefined criteria of a fold change >2 or <0.5 and a *p* value < 0.05, we identified 111 DEPs in patients with AAA compared with HCs, of which 86 were upregulated and 25 were downregulated. In this study, these 111 DEPs were used for downstream bioinformatic analyses. FDR-adjusted *p* values for these DEPs are also provided in Supplementary Table S1, and the DEPs were visualized using volcano plots (Figure 2B). GO and KEGG enrichment analyses were performed to further clarify the biological implications of the DEPs. In the biological process category, the most enriched GO terms were related to the oxidative stress response, reactive oxygen species metabolism, vascular remodeling, and endothelial proliferation. Additional terms included mitochondrial respiration, cellular respiration, oxidative phosphorylation, and electron transport chain activity. The cellular component terms were primarily related to mitochondrial respiratory chain complexes and mitochondrial compartments (Figure 2C). Consistent with these findings, KEGG pathway analysis revealed that the DEPs were enriched in oxidative phosphorylation, reactive oxygen species-related stress responses, tumor necrosis factor signaling, and atherosclerosis-associated pathways (Figure 2D). Taken together, these results suggest that plasma proteomic alterations in patients with AAA are closely associated with mitochondrial metabolic dysfunction, oxidative stress, and inflammatory activation, all of which are key pathophysiological processes involved in AAA pathogenesis.

### 2.3. AAA-Associated DEPs Were Enriched in Mitochondrial Respiratory Network Modules

**Figure 3:**
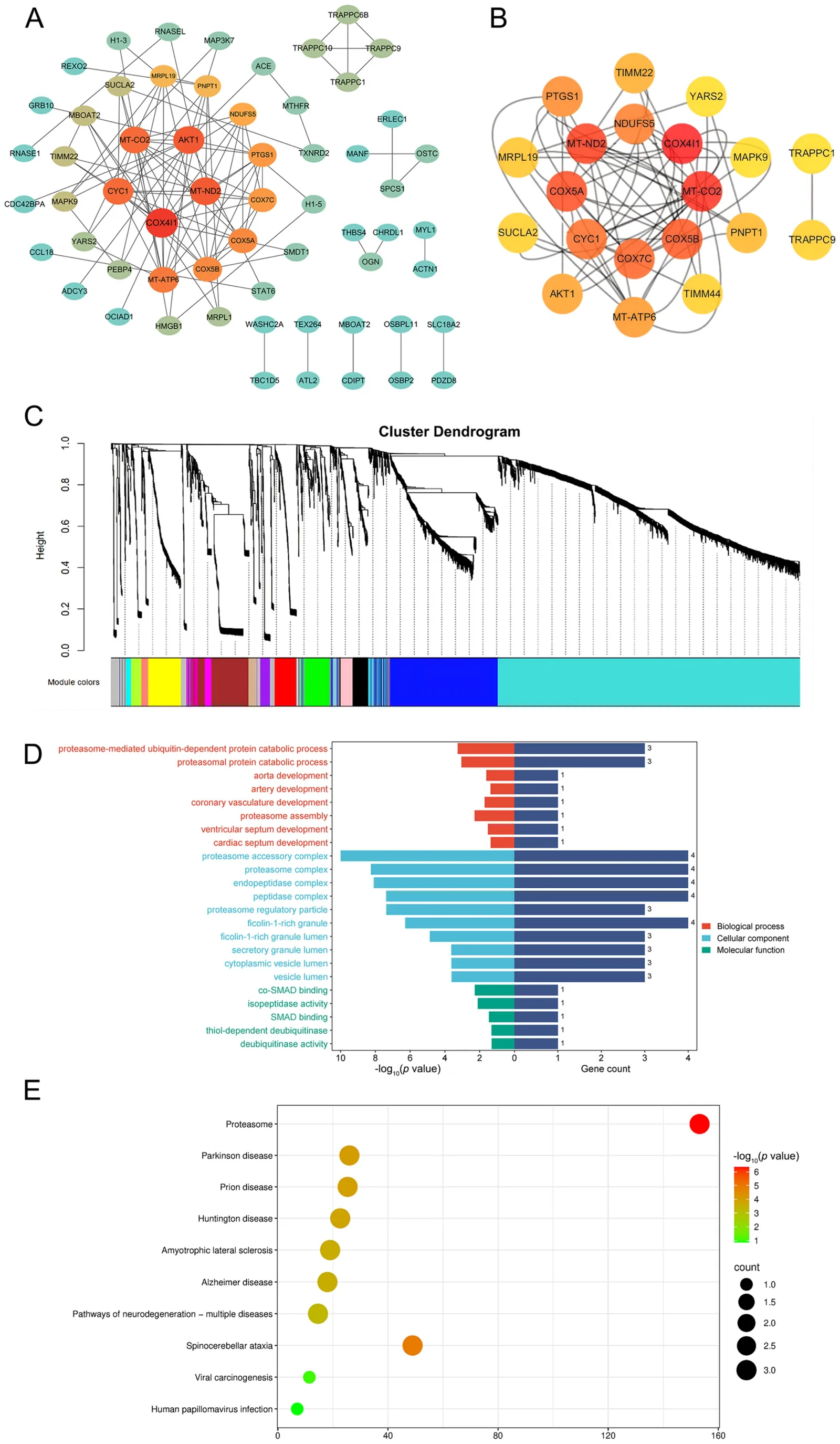
PPI network analysis and WGCNA based on quantified proteins. (**A**) Protein–protein interaction (PPI) network of DEPs between AAA patients and HCs. The nodes represent proteins, and the edges represent protein–protein interactions. Node colors indicate the ranking scores of proteins, with warmer colors representing higher ranking scores. (**B**) Hub proteins identified from the PPI network. The node size and color indicate the ranking score of the hub proteins. (**C**) Cluster dendrogram of weighted gene coexpression network analysis (WGCNA) based on all quantified proteins in the DIA proteomic dataset. The color bar below the dendrogram represents the assigned coexpression modules. (**D**) GO enrichment analysis of proteins in the selected WGCNA module, including the biological process, cellular component, and molecular function categories. (**E**) KEGG pathway enrichment analysis of proteins in the selected WGCNA module. The bubble size represents the number of enriched proteins, and the color indicates the significance level expressed as −log_10_(*p* value).

A protein–protein interaction (PPI) network was constructed using the STRING database and visualized in Cytoscape to investigate the potential interactions among the DEPs. The resulting network contained 111 nodes and 109 edges, with an average node degree of 1.96 and an average local clustering coefficient of 0.389 (Figure 3A). The PPI enrichment *p* value was 4.19 × 10^−7^, indicating that the proteins in this network had significantly more interactions than expected by chance. These findings suggest that the DEPs are functionally connected and may participate in coordinated biological processes during AAA progression.

To further identify the central proteins within the PPI network, the hub proteins were screened using CytoHubba in Cytoscape (Figure 3B). Most of these hub proteins were concentrated in a densely connected mitochondrial respiratory chain-related cluster, suggesting that mitochondrial function and energy metabolism represent key biological features associated with AAA. Among them, COX4I1, MT-ND2, and other hub proteins were closely linked to mitochondrial electron transport and oxidative phosphorylation, indicating that mitochondrial respiratory dysfunction may be involved in AAA progression.

### 2.4. Coexpression Network Analysis Identified a Proteasome-Related AAA Module

Weighted gene coexpression network analysis (WGCNA) was performed to identify AAA-associated plasma protein coexpression modules. On the basis of hierarchical clustering and dynamic tree cutting, multiple coexpression modules were identified from the quantitative proteomic dataset (Figure 3C). Among these modules, the green module was associated with AAA status and was selected for further functional characterization.

To explore the biological features of the AAA-associated green module, enrichment analysis was performed on the top-ranked proteins within this module. GO analysis revealed that these proteins were enriched mainly in proteasome-mediated ubiquitin-dependent protein catabolic processes, proteasomal protein catabolic processes, proteasome assembly, blood vessel development, and aortic development (Figure 3D). KEGG pathway analysis further identified the proteasome pathway as the most prominently enriched pathway in the green module (Figure 3E). Together, these findings indicate that AAA-associated changes in coexpression may involve altered proteasome activity, ubiquitin-dependent protein turnover, and vascular wall remodeling, providing module-level information complementary to the results of global DEP and PPI analyses.

### 2.5. CHRDL1, OGN, and CCL18 Were Validated as Promising Plasma Biomarkers for AAA

**Figure 4:**
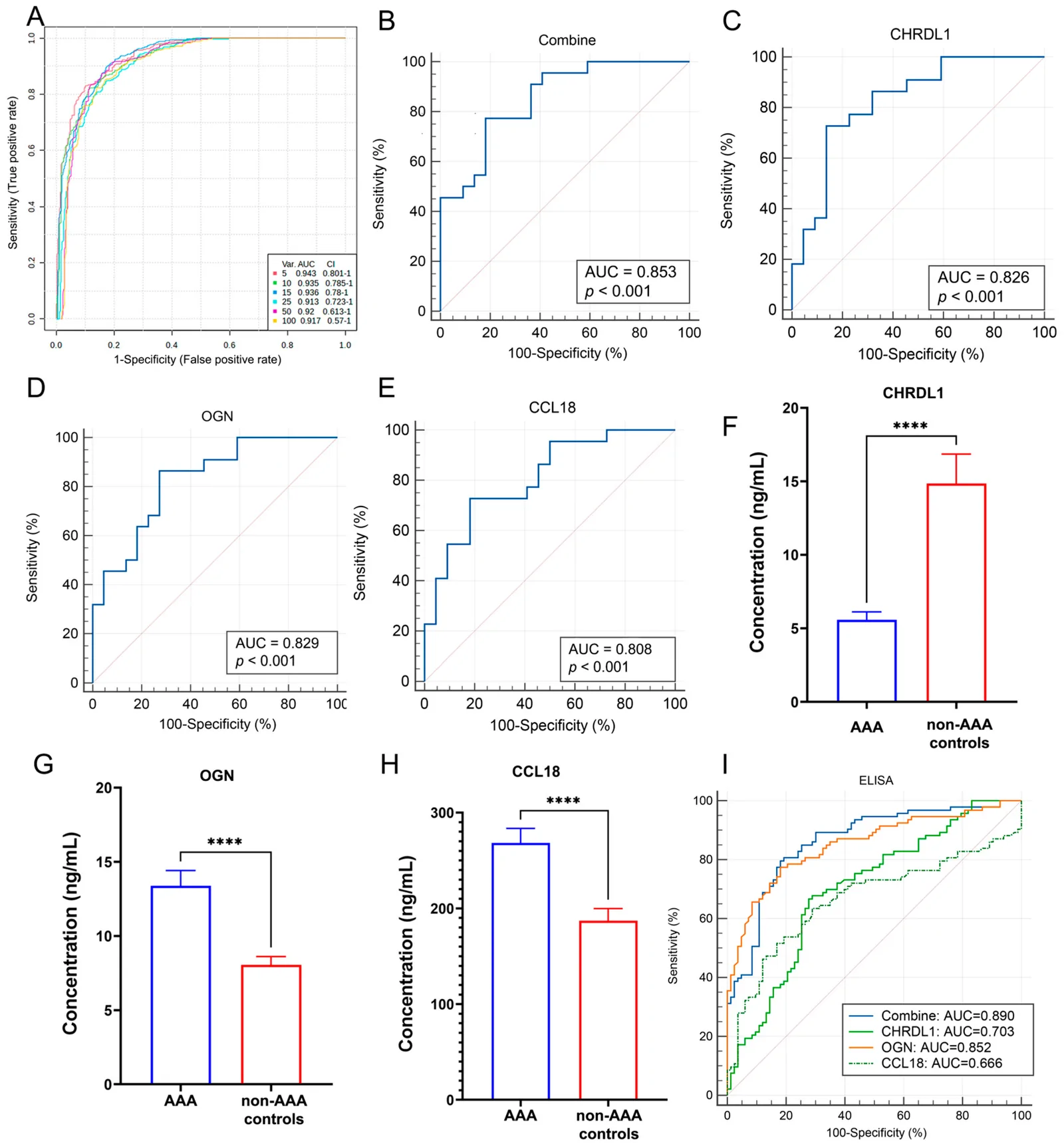
ROC analysis and ELISA validation of candidate plasma biomarkers for AAA. (**A**) Receiver operating characteristic (ROC) curves of combined biomarker models containing different numbers of proteins selected from among the DEPs. (**B**) ROC curve of the combined model based on CHRDL1, OGN, and CCL18 identified in the discovery analysis. (**C**–**E**) ROC curves of individual candidate biomarkers: (**C**), CHRDL1; (**D**), OGN; (**E**), CCL18. (**F**–**H**) Plasma concentrations of candidate biomarkers measured by enzyme-linked immunosorbent assay (ELISA) in AAA patients and non-AAA controls: (**F**), CHRDL1; (**G**), OGN; (**H**), CCL18. (**I**) ROC curve of the combined ELISA-based model including CHRDL1, OGN, and CCL18. The data are presented as the mean ± SEM. **** *p* < 0.0001.

To identify potential diagnostic biomarkers for AAA, Receiver Operating Characteristic (ROC) analysis was performed on the 111 DEPs. We first compared the predictive performance of biomarker panels containing different numbers of proteins. The combined models showed consistently high discriminatory ability, with the five-protein panel achieving the highest area under the curve (AUC) of 0.943 (Figure 4A). Considering their diagnostic performance, biological relevance, and potential clinical applicability, CHRDL1, OGN, and CCL18 were selected as candidate biomarkers for further evaluation.

The combined model based on CHRDL1, OGN, and CCL18 showed a good ability to distinguish patients with AAA from controls, with an AUC of 0.853 (95% CI: 0.714–0.942) (Figure 4B). When evaluated individually, CHRDL1, OGN, and CCL18 also demonstrated diagnostic potential, with AUC values of 0.835, 0.837, and 0.813, respectively (Figure 4C–E).

To further validate these candidate biomarkers, plasma levels of CHRDL1, OGN, and CCL18 were measured by ELISA in an independent validation cohort. The ELISA results were consistent with the proteomic findings: compared with controls, the CHRDL1 abundance significantly decreased in patients with AAA, whereas the OGN and CCL18 abundance significantly increased in patients with AAA (Figure 4F–H). Furthermore, the combined diagnostic model of the three proteins achieved an AUC of 0.890 (95% CI: 0.826–0.938) for discriminating patients with AAA from non-AAA controls (Figure 4I). These findings support CHRDL1, OGN, and CCL18 as a complementary three-protein biomarker panel with potential diagnostic value for AAA.

### 2.6. Virtual Knockout Analysis Suggested Distinct Functional Roles for Three Candidate Biomarkers in AAA

To further clarify the cellular origins and potential functions of CHRDL1, OGN, and CCL18 in AAA, we analyzed a public scRNA-seq dataset (GSE166676 and GSE226492). After quality control, dimensionality reduction, clustering, and cell type annotation, major aortic cell populations, including smooth muscle cells, fibroblasts, macrophages, and endothelial cells, were identified. CHRDL1 and OGN were expressed primarily in fibroblasts. In contrast, CCL18 was predominantly expressed in macrophages. To explore their potential functional roles, we performed in silico knockout analysis using scTenifoldKnk. CHRDL1, OGN, and CCL18 were virtually knocked out in the major cell populations expressing these genes. The resulting perturbation profiles were then subjected to GO and KEGG enrichment analyses.

**Figure 5:**
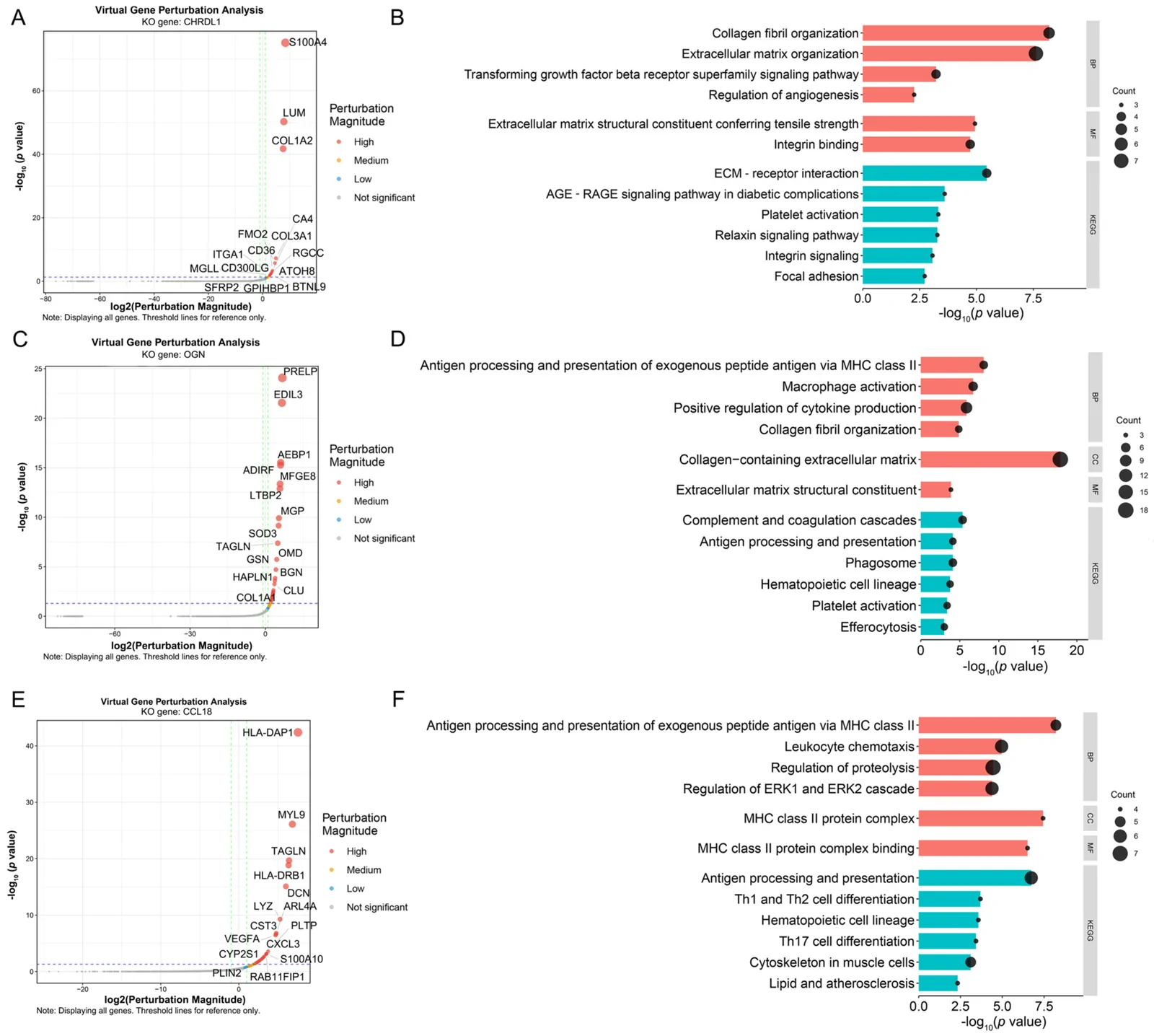
Single-cell-based virtual gene perturbation analysis of CHRDL1, OGN, and CCL18. (**A**) Virtual gene perturbation analysis after CHRDL1 knockout. (**B**) GO and KEGG enrichment analyses of genes whose expression was perturbed after CHRDL1 knockout, including genes associated with the biological process, cellular component, and molecular function categories and enriched pathways. (**C**) Virtual gene perturbation analysis after OGN knockout. (**D**) GO and KEGG enrichment analyses of genes whose expression was perturbed after OGN knockout, including genes associated with the biological process, cellular component, and molecular function categories and enriched pathways. (**E**) Virtual gene perturbation analysis after CCL18 knockout. (**F**) GO and KEGG enrichment analyses of genes whose expression was perturbed after CCL18 knockout, including genes associated with the biological process, cellular component, and molecular function categories and enriched pathways. In (**A**,**C**,**E**), the labeled points indicate representative perturbed genes, the dashed horizontal line indicates the significance threshold, and the dashed vertical lines indicate the perturbation magnitude thresholds. In (**B**,**D**,**F**), different colors represent different functional categories (GO categories or KEGG pathways), and the bar length represents the enrichment significance expressed as −log_10_(*p* value).

Virtual knockout of CHRDL1 affected mainly ECM-related genes, including S100A4, LUM, and COL1A2 (Figure 5A). GO analysis unveiled that CHRDL1-related genes were enriched primarily in biological processes associated with collagen fibril organization, ECM organization, and epithelial-to-mesenchymal transition, as well as in molecular functions related to ECM structural constituents and collagen binding. KEGG analysis further highlighted the ECM–receptor interaction, AGE–RAGE signaling, platelet activation, relaxin signaling, and integrin signaling pathways (Figure 5B). These findings suggest that CHRDL1 may participate in fibroblast/mesenchymal cell-mediated ECM remodeling and matrix–vascular signaling in AAA.

After OGN was knocked out, the expression profiles of several matrix- and stromal cell-associated genes, including PRELP, EDIL3, AEBP1, MFGE8, and MGP, were strongly perturbed (Figure 5C). GO enrichment analysis indicated that OGN-related perturbations were associated with macrophage activation, leukocyte activation, positive regulation of cytokine production, collagen fibril organization, and ECM structural constituents. KEGG analysis further revealed enrichment in the complement and coagulation cascades, antigen processing and presentation, phagosome, hematopoietic cell lineage, and platelet activation pathways (Figure 5D). These results suggest that OGN may contribute not only to stromal matrix remodeling but also to immune- and coagulation-related regulation in AAA.

With respect to CCL18, virtual knockout affected mainly immune-related genes, including HLA-DPA1, MYL9, TAGLN, HLA-DRB1, and DCN (Figure 5E). GO enrichment analysis showed that CCL18-related perturbations were involved predominantly in antigen processing and presentation via MHC class II, leukocyte chemotaxis, regulation of proteolysis, regulation of the ERK1/2 cascade, and MHC class II protein complex-related functions. KEGG analysis further identified antigen processing and presentation, Th1 and Th2 cell differentiation, hematopoietic cell lineage, Th17 cell differentiation, and lipid and atherosclerosis pathways (Figure 5F). These findings indicate that CCL18 may be involved in macrophage-mediated antigen presentation, leukocyte recruitment, and adaptive immune regulation in AAA.

## 3. Discussion

AAA is a degenerative vascular disease with an insidious clinical course and a high mortality rate after rupture [3,4]. At present, imaging modalities such as ultrasound and CTA remain the main tools for AAA detection and surveillance, whereas reliable circulating biomarkers for early diagnosis are still lacking [6,7]. In recent years, several plasma biomarkers have been proposed for the clinical assessment of AAA [20,21,22,23]. However, their diagnostic performance and clinical applicability remain insufficiently established. In the present study, DIA-based plasma proteomics identified distinct systemic protein alterations associated with AAA and revealed a biomarker panel composed of CHRDL1, OGN, and CCL18 with promising diagnostic performance. These findings provide additional insight into the molecular mechanisms underlying AAA and support the potential use of multimarker plasma signatures for noninvasive disease detection.

A major strength of this study is the integration of unbiased proteomic discovery with independent experimental validation. Recent AAA biomarker studies have broadened the field through plasma proteomics, extracellular vesicle proteomics, tissue-resolved proteomics, and proteome-wide causal inference analyses [24,25,26,27]. Notably, several proteins identified among our DEPs, including RNASE4, ACTN1, PLOD3, THBS4, and HMGB1, have also been implicated in recent AAA-related studies [28,29,30,31,32,33]. These proteins are functionally linked to immune-inflammatory activation, vascular remodeling, and extracellular matrix organization, which is consistent with our findings. Recent AAA biomarker studies have likewise highlighted convergent biological themes, including inflammatory signaling, macrophage-associated immune remodeling, extracellular matrix turnover, and protease-related pathways [24,25,26,32,33]. These observations are broadly consistent with our findings that chronic inflammation and ECM remodeling are central features of AAA biology. In addition to these established features, our integrated DIA-based analysis further highlighted mitochondrial dysfunction and proteostasis-related alterations, suggesting the presence of additional layers of systemic plasma remodeling in AAA. In this context, our study provides a discovery-to-validation workflow combining unbiased plasma proteomics, bioinformatics analysis, protein biomarker panel selection, and independent ELISA validation in a clinically accessible format.

Integrated functional analyses of the DEPs consistently suggested that mitochondrial dysfunction, oxidative stress, inflammatory activation, ECM remodeling, and dysregulated proteostasis are major biological features associated with AAA. GO and KEGG enrichment analyses unveiled significant alterations in oxidative phosphorylation, mitochondrial respiration, reactive oxygen species-related pathways, and vascular remodeling processes, suggesting that systemic metabolic stress and inflammatory activation are involved in AAA. Consistent with this, the results of the PPI network analysis identified multiple mitochondrial respiratory chain-associated hub proteins, further supporting the potential involvement of impaired energy metabolism and mitochondrial homeostasis in aneurysmal degeneration [34,35,36]. In addition, WGCNA revealed a disease-associated coexpression module enriched in proteasome-mediated protein degradation and ubiquitin-dependent protein turnover pathways, indicating that disturbed proteostasis regulation may also contribute to vascular remodeling and stress responses during AAA progression. Previous studies have demonstrated that mitochondrial dysfunction and oxidative stress can promote vascular smooth muscle cell apoptosis, ECM degradation, inflammatory activation, and vascular wall weakening, whereas dysregulated ubiquitin–proteasome activity has been implicated in vascular aging and maladaptive remodeling [37]. Taken together, these findings suggest that AAA-associated plasma proteomic alterations may reflect coordinated disturbances in mitochondrial metabolism, inflammatory signaling, ECM homeostasis, and protein quality control.

CHRDL1 emerged as one of the most discriminative biomarkers identified in this study. It is a secreted antagonist of bone morphogenetic protein (BMP) signaling and has been implicated in mesenchymal cell differentiation, ECM homeostasis, and tissue remodeling [38]. BMP signaling is known to regulate vascular smooth muscle cell phenotypic switching, osteogenic differentiation, vascular calcification, and ECM turnover, which are closely involved in AAA pathogenesis [39]. Therefore, reduced CHRDL1 expression may reflect impairments in the regulation of BMP-dependent vascular repair and structural homeostasis during aneurysmal progression. In addition, ECM degradation and loss of medial integrity are central pathological features of AAA. Our perturbation analyses further support a potential association between CHRDL1 and ECM organization, suggesting that CHRDL1 participates in maintaining vascular structural homeostasis.

OGN, a small leucine-rich ECM proteoglycan, was also significantly elevated in AAA plasma samples. Previous studies have shown that OGN is involved in collagen fibrillogenesis, matrix organization, and tissue fibrosis [40]. Progressive degradation and disorganization of collagen and elastin fibers are key pathological hallmarks of AAA; thus, increased levels of circulating OGN may reflect an active but potentially insufficient compensatory remodeling response within the aneurysmal wall [41]. In addition to the structural role of OGN in matrix organization, accumulating evidence suggests that ECM components can actively regulate inflammatory signaling and vascular cell behavior. Our analyses further demonstrated that OGN-associated pathways were linked to not only matrix remodeling but also immune/coagulation-related processes, suggesting that OGN participates in stromal–immune cell signaling during AAA progression. Because vascular remodeling in AAA is closely coupled with chronic inflammation and thrombus-associated biological activity, elevated OGN may reflect coordinated matrix repair, stromal activation, and inflammatory remodeling responses within the diseased aortic wall.

CCL18 levels were also elevated in AAA plasma and showed potential diagnostic value. CCL18 is predominantly secreted by alternatively activated macrophages and is associated with chronic inflammation, fibrosis, and tissue remodeling in multiple inflammatory diseases [42]. Macrophage infiltration is a hallmark of AAA lesions and contributes to chronic inflammation, protease secretion, ECM degradation, and vascular wall weakening [43]. Persistent macrophage activation can further promote cytokine release and inflammatory cell recruitment, thereby sustaining a chronic inflammatory microenvironment within the aneurysmal wall. Therefore, elevated circulating CCL18 levels may reflect macrophage-driven immune activation and chronic inflammatory remodeling during AAA progression. Collectively, CHRDL1, OGN, and CCL18 may reflect complementary but interconnected pathological dimensions of AAA, including ECM destabilization, stromal remodeling, maladaptive vascular repair, and chronic immune activation. These findings support the concept that AAA progression is driven not only by a single pathological pathway but also by coordinated interactions among vascular structural degeneration, inflammatory amplification, and dysregulated tissue remodeling.

Taken together, our findings support a model in which AAA-associated vascular wall injury, mitochondrial dysfunction, oxidative stress, ECM remodeling, immune activation, and dysregulated proteostasis collectively contribute to the alterations observed in the circulating plasma proteome. Integrated bioinformatic analyses further suggested that mitochondrial respiratory dysfunction, oxidative phosphorylation imbalance, and disturbed ubiquitin–proteasome activity are major molecular features associated with AAA. These findings provide a mechanistic context for validated plasma biomarkers and support the biological rationale for combining CHRDL1, OGN, and CCL18 into a predictive biomarker panel.

From a biological perspective, CHRDL1, OGN, and CCL18 may reflect distinct but complementary pathological processes in AAA. Clinically, compared with individual proteins alone, the combined biomarker model demonstrated superior diagnostic performance, supporting the potential value of multimarker strategies for AAA detection. Notably, all three proteins were successfully validated by ELISA in an independent cohort, supporting their reproducibility and technical feasibility as circulating biomarkers.From a clinical perspective, the proposed biomarker panel can be quantified through blood-based assays, offering advantages such as convenient operation and minimal invasiveness. After further prospective validation, this panel may have value in several clinically relevant scenarios. For example, because AAA screening is clinically considered in older adults, particularly men aged more than 65 years with a history of smoking, family history of AAA or other vascular risk factors [44,45], this blood-based panel may provide a practical tool for large-scale preliminary risk assessment in health examination settings or public screening programs. In addition, among patients who present to cardiovascular or vascular services, especially those with coexisting cardiocerebrovascular disease, this panel may offer a rapid and accessible method for identifying individuals who could benefit from further targeted treatment. This may be particularly relevant for patients in whom CTA carries higher risk, such as those with impaired renal function, for whom a blood-based strategy could provide a useful approach for assessment. Therefore, from a translational perspective, this multimarker strategy may have potential utility as a future screening or triage tool, but its real-world clinical value still requires confirmation in prospective and representative populations.

This study has several limitations. First, this was a single-center, cross-sectional study. Although the candidate biomarkers were validated in an independent cohort, larger multicenter studies including diverse populations are needed to confirm the reproducibility and clinical applicability of the CHRDL1, OGN, and CCL18 biomarker panel. Second, the present study focused mainly on the diagnostic discrimination of patients with AAA from non-AAA controls and did not evaluate the association of these biomarkers with rupture risk or outcomes. Thus, prospective longitudinal studies with serial plasma sampling and imaging follow-up are needed to determine whether these biomarkers can dynamically reflect rupture-related risk. Third, the functional implications of CHRDL1, OGN, and CCL18 were inferred from single-cell data and in silico knockout analysis. Further experimental validation using vascular cells, aneurysmal tissue specimens, and animal models is necessary to define the mechanistic role of these cells in AAA pathogenesis.

## 4. Materials and Methods

### 4.1. Study Design

This cross-sectional study was conducted in accordance with the Strengthening the Reporting of Observational Studies in Epidemiology (STROBE) guidelines. The study protocol was approved by the Institutional Review Board of PUMCH. Written informed consent was obtained from all participants before enrollment, and all procedures were performed in accordance with institutional regulations and the Declaration of Helsinki.

The study consisted of a proteomic discovery phase followed by an experimental validation phase. In the discovery phase, plasma samples from patients with AAA and healthy controls (HCs) were analyzed using DIA-based proteomics to identify DEPs associated with AAA. Bioinformatic analyses were subsequently performed to characterize the biological functions and pathways related to these DEPs. Candidate plasma biomarkers were selected on the basis of differential abundance, ROC analysis, and biological relevance. In the validation phase, ELISA was used to quantify the levels of candidate proteins in an independent plasma cohort of AAA patients and controls. The overall aim of this discovery-to-validation workflow was to identify novel plasma biomarkers capable of distinguishing AAA patients from non-AAA controls.

### 4.2. Study Population

The AAA patients included both patients with small aneurysms from the clinic and patients with large aneurysms that indicated the need for surgery at the Department of Vascular Surgery, PUMCH. The diagnosis of AAA was confirmed by abdominal imaging, primarily CTA, and individuals whose maximum abdominal aortic diameter was 30 mm or greater were considered eligible. Patients were excluded if they had inflammatory AAA, dissecting aneurysms, pseudoaneurysms, ruptured AAA, aortitis, malignancy, or other severe systemic diseases that could substantially affect plasma protein profiles. For all the enrolled patients, demographic characteristics, medical history, laboratory test results, imaging findings, and treatment information were systematically recorded.

During the same period, HCs in the discovery cohort were recruited from the PUMCH Physical Examination Center and inpatient wards and were selected to match patients with AAA by age and sex as much as possible.

The validation cohort was also derived from consecutive outpatients and inpatients in the Department of Vascular Surgery of PUMCH during the study period. Patients with AAA in the validation cohort were consecutively enrolled after the diagnosis was clinically confirmed. The baseline clinical characteristics, inclusion criteria, and exclusion criteria of the validation cohort were kept consistent with those of the discovery cohort as much as possible. Non-AAA controls were defined as outpatients and inpatients from PUMCH in whom AAA was excluded through imaging evaluation. Individuals with infectious diseases, rheumatic or autoimmune disorders, malignancy, clinically evident vascular disease, or aortic aneurysmal disease were excluded from the non-AAA controls group. Written informed consent was obtained from all the participants before sample collection.

For the proteomic discovery phase, plasma samples from 22 patients with AAA and 22 HCs were subjected to DIA-based proteomic analysis. For the validation phase, an independent cohort consisting of 93 patients with AAA and 83 non-AAA controls was used to validate the candidate biomarkers by ELISA.

### 4.3. Plasma Collection and Preparation

Following an overnight fast of at least 8 h, venous blood was collected from participants in the morning into multiple tubes containing ethylenediaminetetraacetic acid. Each tube was gently inverted 10 times to thoroughly mix the blood with the anticoagulant. The plasma was then isolated by centrifugation at 2000 rpm for 10 min at 4 °C, transferred to sterile Eppendorf tubes, and stored at −80 °C until protein extraction.

Each 100 μL plasma sample was mixed with magnetic covalent organic framework particles and incubated at 37 °C for 60 min. The magnetic particles were subsequently washed 3 times with washing buffer and mixed with lysis buffer. The mixture was then subjected to reduction and alkylation at 95 °C for 10 min. After cooling to room temperature, each mixture was digested with trypsin at 37 °C for 2 h, after which 0.1% trifluoroacetic acid buffer was added to stop digestion. The peptide mixtures were then desalted and dried by vacuum evaporation.

### 4.4. DIA Proteomic Analysis

The peptides derived from the plasma samples were reconstituted in 0.1% formic acid and prepared for proteomics analysis. For each sample, 1 μg of the peptides was injected and separated using an UltiMate^™^ 3000 liquid chromatography system (Thermo Scientific, Bremen, Germany) coupled to an Orbitrap Exploris 480 (Thermo Fisher Scientific, Waltham, MA, USA) mass spectrometer through an EASY-Spray ion source. The peptide samples were separated on an analytical column (C18, 75 μm × 500 mm; Kyoto Monotech, Kyoto, Japan) with a 60-min elution procedure. Mobile phase A consisted of 99.9% H_2_O and 0.1% formic acid, whereas mobile phase B consisted of 80% acetonitrile, 19.9% H_2_O and 0.1% formic acid. Peptides were separated using a multistep increasing organic gradient, followed by high-organic washing and column re-equilibration. The electrospray voltage was set to 2.3 kV, and the capillary temperature was maintained at 320 °C.

Mass spectrometry data were acquired in DIA mode. Each acquisition cycle started with a full MS scan over the m/z range of 350–1200 at a resolution of 120,000, with an automatic gain control target of 3 × 10^6^ ions. This was followed by DIA MS/MS scans using 80 isolation windows at a resolution of 30,000. Precursor ions were fragmented by higher-energy collisional dissociation using stepped normalized collision energies of 25%, 30%, and 35%. For the DIA MS/MS scans, the automatic gain control target was set to 2 × 10^5^ ions, and the maximum injection time was 50 ms.

### 4.5. Mass Spectrometry Data Processing

The DIA spectral library was generated from the raw mass spectrometry data using Spectronaut software (version 17.2). A data search was conducted against the human UniProt database (2026 release, containing 20,405 protein entries) with a parent ion tolerance of 10 ppm and a fragment ion mass tolerance of 0.05 Da. Trypsin/P was selected as the digestion enzyme, and up to two missed cleavages were allowed. Carbamidomethylation of cysteine was specified as a fixed modification, whereas methionine oxidation and protein N-terminal acetylation were set as variable modifications. The false discovery rate (FDR) thresholds for peptide–spectrum matches, peptides, and protein groups were all controlled below 1%. The quantification of peptide intensity was based on the sum of the peak areas of the fragment ions in MS2. Protein abundance matrices exported from Spectronaut were used for subsequent differential abundance and bioinformatic analyses. A local regression method was applied to normalize the cross-run data, and proteins were filtered using a Q value threshold of 0.01. Variables absent in more than 50% of the samples were removed from further statistical analysis, and the remaining missing values were imputed using the K-nearest neighbor (KNN) method. Differential protein abundance analysis was then performed using the limma package with empirical Bayes moderation. Proteins with a fold change >2 or <0.5 and a *p* value < 0.05 were defined as significantly differentially abundant proteins.

### 4.6. Functional Analysis

A volcano plot was generated using an online bioinformatics platform (https://www.bioinformatics.com.cn, accessed on 10 August 2026), which was used for data visualization and analysis. PLS-DA was conducted using SIMCA software (version 15.0). Functional annotation using Gene Ontology (GO) and InterPro (IPR) was performed with the InterProScan program, which incorporates databases. Protein families and pathways were examined through Clusters of Orthologous Groups and Kyoto Encyclopedia of Genes and Genomes (KEGG) analysis. Functional enrichment analyses were performed on the basis of the DEPs identified from the DIA proteomic analysis, using the whole human genome as the background set. GO and KEGG pathway enrichment analyses were performed to characterize the biological functions and pathways associated with AAA-related protein alterations.

### 4.7. PPI Network Analysis

PPI network analysis was performed to explore the potential interactions among the DEPs and to identify key molecular modules associated with AAA. The list of DEPs was uploaded to the Search Tool for the Retrieval of Interacting Genes/Proteins (STRING, https://cn.string-db.org/, version 12.0) database to construct the PPI network. Interactions with confidence scores above the predefined threshold were retained for further analysis. The resulting network was imported into Cytoscape software (version 3.10.3) for visualization and topological analysis. Hub proteins were screened using the CytoHubba (version 0.1) plugin on the basis of commonly used topological algorithms. A functional enrichment analysis of the top genes was subsequently performed to characterize their biological significance.

### 4.8. WGCNA

WGCNA was performed using all the proteins quantified in the DIA proteomic dataset after quality control. The use of the complete quantified protein set allowed unbiased identification of coexpression modules associated with AAA status. The quantitative proteomic abundance matrix was used as the input data. After proteins with excessive missing values or low abundance were filtered, a coexpression network was constructed using the WGCNA package in R software (version 4.5.3). An appropriate soft-thresholding power was selected to achieve approximate scale-free topology. Proteins were then clustered into modules on the basis of topological overlap similarity. Module eigengenes were calculated and correlated with clinical traits, including AAA status. Modules strongly associated with AAA were considered disease-related modules. Hub proteins within key modules were further identified according to module membership and protein significance. A functional enrichment analysis was performed to explore the biological processes and pathways represented by the key modules.

### 4.9. ROC Analysis

ROC analysis was performed to evaluate the diagnostic performance of the candidate plasma biomarkers for distinguishing patients with AAA from controls. For each DEP, ROC curves were generated, and the AUC was calculated with corresponding 95% confidence intervals. To further prioritize candidate biomarkers, machine learning-based multivariate exploratory ROC models were constructed and compared. The proteins contributing most to the best-performing model were ranked by average variable importance, and the top 15 proteins were selected as candidate biomarkers for further evaluation. These candidates were subsequently prioritized based on their associations with AAA, potential biological relevance to AAA pathogenesis, and feasibility for downstream experimental validation. In particular, the availability of compatible commercial ELISA kits was considered to facilitate independent validation. Based on this integrated evaluation combining machine-learning prioritization, disease relevance, and experimental accessibility, CHRDL1, OGN, and CCL18 were ultimately selected to establish the three-protein biomarker panel. In the validation cohort, ROC curve analysis was repeated using ELISA-measured plasma protein concentrations. For the combined biomarker models, binary logistic regression was used to generate predicted probabilities, which were subsequently used to construct ROC curves. Sensitivity, specificity, and optimal cutoff values were determined according to the Youden index. Statistical and ROC curve analyses were performed using MedCalc 23.4.0 and GraphPad Prism 9.

### 4.10. ELISA Validation of Several Promising Biomarkers

A double-antibody sandwich ELISA was used to validate proteins in 176 plasma samples (93 from patients with AAA and 83 from non-AAA controls) according to the manufacturer’s recommended protocol. ELISA kits (Cusabio, Wuhan, China) for the following proteins were used: CHRDL1 (Chordin-like 1), OGN (Osteoglycin), and CCL18 (C-C motif chemokine ligand 18). Preliminary experiments determined the optimal dilution factor for plasma samples. Plates were read at 450 nm and 630 nm using a microplate reader (ELx800NB, BioTek, Winooski, VT, USA). All protein assays revealed less than 10% intraplate variation. GraphPad Prism 9 was used for graphing.

### 4.11. In Silico Knockout Validation Using Public Single-Cell Data

To further explore the potential functions of the validated biomarkers, we downloaded a single-cell RNA sequencing (scRNA-seq) dataset for AAA from the GEO database (GSE166676 and GSE226492). After standard quality control, dimensionality reduction, clustering, and cell type annotation, we extracted the target cell subpopulations. In silico knockout of the target genes was performed using scTenifoldKnk software (version 1.0.3). By comparing the gene regulatory networks before and after knockout, we identified perturbed genes and pathways. The number of permutation tests was set to 1000, and the significance threshold was set at adjusted *p* < 0.05.

### 4.12. Statistical Analysis

The normality of all the quantitative data was assessed using the Shapiro–Wilk test. Normally distributed data are expressed as the mean ± standard deviation and were compared using a two-sided Student’s t test. For skewed data, values are presented as medians (interquartile ranges) and were analyzed with the Wilcoxon rank-sum test. Categorical variables were examined using the chi-square test or Fisher’s exact test, as appropriate. Spearman correlation analysis was performed to evaluate the relationships between the DEPs and clinical parameters. A *p* value <0.05 was considered to indicate statistical significance. Unless otherwise indicated, all the statistical analyses were performed using R software (version 4.5.3) and SPSS software (version 26).

## 5. Conclusions

In this study, DIA-based plasma proteomics, bioinformatics network analysis, ROC-based candidate selection, ELISA validation, and single-cell-based exploratory analysis were integrated to identify a circulating biomarker panel consisting of CHRDL1, OGN, and CCL18 with promising diagnostic potential for AAA. Integrated proteomic and bioinformatic analyses further suggested that mitochondrial dysfunction, ECM remodeling, and chronic inflammation are closely associated with AAA pathogenesis. These findings provide additional insight into the molecular landscape of AAA and may facilitate the future development of noninvasive biomarkers and therapeutic targets for AAA.

## Figures and Tables

**Table 1:** Baseline characteristics of participants in the proteomic discovery and ELISA validation cohorts.

Items	Proteome Analysis Cohort			ELISA Validation Cohort		
	AAA (*n* = 22)	HC (*n* = 22)	*p*-Value	AAA (*n* = 93)	non-AAA Controls (*n* = 83)	*p*-Value
Age (years)	70.9 ± 8.7	66.1 ± 7.9	0.062	70.5 ± 8.4	68.7 ± 7.6	0.145
Male	21 (95.5)	17 (77.3)	0.188	71 (76.3)	63 (75.9)	0.945
Smoke	15 (68.2)	12 (54.5)	0.353	53 (57.0)	38 (45.8)	0.138
Hypertension	17 (77.3)	15 (68.2)	0.498	72 (77.4)	63 (75.9)	0.812
Diabetes Mellitus	2 (9.1)	6 (27.3)	0.241	11 (11.8)	18 (21.7)	0.078
Chronic Kidney Disease	1 (4.5)	4 (18.2)	0.342	8 (8.6)	3 (3.6)	0.172
COPD	1 (4.5)	0 (0.0)	1.000	2 (2.2)	5 (6.0)	0.592
Baseline AAA maximal diameter (mm)	55.3 ± 18.1	20.4 ± 3.5	<0.001 *	62.8 ± 16.8	22.1 ± 3.3	<0.001 *

The data are presented as the mean ± standard deviation for continuous variables and as the number (%) for categorical variables. Comparisons between AAA patients and HCs were performed using Student’s t test or the Wilcoxon rank-sum test for continuous variables and the chi-square test or Fisher’s exact test for categorical variables, as appropriate. * *p* < 0.05 was considered to indicate statistical significance. AAA, abdominal aortic aneurysm; HC, healthy control; ELISA, enzyme-linked immunosorbent assay; COPD, chronic obstructive pulmonary disease.

## Data Availability

The mass spectrometry proteomics data have been deposited in the ProteomeXchange Consortium (https://proteomecentral.proteomexchange.org, accessed on 10 August 2026) via the iProX partner repository with the dataset identifier PXD079983.

## Supplementary Materials

The following supporting information can be downloaded at: https://www.mdpi.com/article/10.3390/ijms27167312/s1.

## Abbreviations

The following abbreviations are used in this manuscript:

AAA	Abdominal aortic aneurysm
AUC	Area under the curve
BMP	Bone morphogenetic protein
CCL18	C-C motif chemokine ligand 18
CHRDL1	Chordin-like 1
COPD	Chronic obstructive pulmonary disease
CTA	Computed tomography angiography
DEPs	Differentially expressed proteins
DIA	Data-independent acquisition
ECM	Extracellular matrix
ELISA	Enzyme-linked immunosorbent assay
FDR	False discovery rate
GO	Gene Ontology
HC	Healthy control
IPR	InterPro
KEGG	Kyoto Encyclopedia of Genes and Genomes
OGN	Osteoglycin
PLS-DA	Partial least squares discriminant analysis
PPI	Protein–protein interaction
scRNA-seq	Single-cell RNA sequencing
STRING	Search Tool for the Retrieval of Interacting Genes/Proteins
WGCNA	Weighted gene coexpression network analysis
