# Supplementary material for: Identification and Validation of Plasma Protein Biomarkers for Abdominal Aortic Aneurysm Using Integrated Proteomics
Source: Int J Mol Sci. 2026 Aug 16;27(16):7312. doi: 10.3390/ijms27167312 (PMC13513117; doi:10.3390/ijms27167312)
Supplement: Supplementary file 1 [file ijms-27-07312-s001.zip › ijms-4441160-supplementary.pdf]

Uniprot	Gene name	Protein description	FC	logFC	p value	adj.p Value	adj.p Value (Clinical confounders)
P12821	ACE	Angiotensin-converting enzyme	40.658	5.345467344	0.036499	0.042744373	0.059183657
A0A7I2V4Y4	ACTN1	Actinin alpha 1	0.48	-1.058893689	0.001204	0.008971759	0.010700808
O60266	ADCY3	Adenylate cyclase type 3	5.138	2.36120689	0.050361	0.048871891	0.029081019
P31749	AKT1	RAC-alpha serine/threonine-protein kinase	6.905	2.787641414	0.01791	0.036029711	0.016683231
Q9UJX3	ANAPC7	Anaphase-promoting complex subunit 7	6.717	2.747817029	0.018411	0.036029711	0.016292152
Q8IV38	ANKMY2	Ankyrin repeat and MYND domain-containing protein 2	85.641	6.420229737	0.007748	0.022984919	0.007648529
Q9Y587	AP4S1	AP-4 complex subunit sigma-1	58.772	5.877057089	0.036823	0.042744373	0.020339775
Q8NHH9	ATL2	Atlastin-2	18.369	4.199201184	0.039617	0.042744373	0.013017788
Q9NWV8	BABAM1	BRISC and BRCA1-A complex member 1	6.355	2.667892125	0.017935	0.036029711	0.048311218
Q86YS7	C2CD5	C2 domain-containing protein 5	5.639	2.495439343	0.033579	0.042744373	0.014715825
Q8N4T8	CBR4	3-oxoacyl-[acyl-carrier-protein] reductase	6.28	2.650764559	0.032238	0.042744373	0.012016572
P55774	CCL18	C-C motif chemokine 18	2.31	1.207892852	0.012202	0.030705232	0.04021885
P13987	CD59	CD59 glycoprotein	27.996	4.807148808	0.038711	0.042744373	0.110056024
Q5VT25	CDC42BPA	Serine/threonine-protein kinase MRCK alpha	55.458	5.793323684	0.036626	0.042744373	0.006371438
O14735	CDIPT	CDP-diacylglycerol--inositol 3-phosphatidyltransferase	0.434	-1.204233052	9.02E-05	0.008169905	0.002774121
Q9Y5P4	CERT1	Ceramide transfer protein	47.467	5.568852967	0.008099	0.022984919	0.00418153
Q9BU40	CHRDL1	Chordin-like protein 1	0.475283	-1.073142293	0.000468	0.01700999	0.013584149
Q9Y240	CLEC11A	C-type lectin domain family 11 member A	26.81	4.744699314	0.038231	0.042744373	0.057582785
O75175	CNOT3	CCR4-NOT transcription complex subunit 3	4.303	2.10534284	0.022581	0.041240854	0.070893133
P13073	COX4I1	Cytochrome c oxidase subunit 4 isoform 1, mitochondrial	0.492	-1.023269779	0.001125	0.022026217	0.015754435
P20674	COX5A	Cytochrome c oxidase subunit 5A, mitochondrial	0.482	-1.052894948	0.001103	0.027402036	0.02436557
P10606	COX5B	Cytochrome c oxidase subunit 5B, mitochondrial	0.497	-1.008682243	0.001982	0.010546903	0.00507601
P15954	COX7C	Cytochrome c oxidase subunit 7C, mitochondrial	0.488	-1.035046947	0.001887	0.019182115	0.011631858
Q96SW2	CRBN	Protein cereblon	35.49	5.149340669	0.036417	0.042744373	0.006464871
P08574	CYC1	Cytochrome c1, heme protein, mitochondrial	0.495	-1.01449957	0.002063	0.027731101	0.022357466
Q9Y394	DHRS7	Dehydrogenase/reductase SDR family member 7	0.457	-1.12973393	0.000265	0.007500224	0.00221834
Q969X5	ERGIC1	Endoplasmic reticulum-Golgi intermediate compartment protein 1	0.484	-1.046921047	6.26E-05	0.008169905	0.003146701
Q96DZ1	ERLEC1	Endoplasmic reticulum lectin 1	30.401	4.926046875	0.008464	0.02337227	0.020828707
Q16394	EXT1	Exostosin-1	2.078	1.055195654	0.000349	0.008105269	0.004006038
Q9UK99	FBXO3	F-box only protein 3	16.445	4.039577103	0.0198	0.037734395	0.021970612
P68106	FKBP1B	Peptidyl-prolyl cis-trans isomerase FKBP1B	2.257	1.174406419	0.039211	0.062891017	0.096976969
Q06787	FMR1	Fragile X messenger ribonucleoprotein 1	7.464	2.899948986	0.01509	0.034113326	0.010701508
Q8TEQ6	GEMIN5	Gem-associated protein 5	21.036	4.394788497	0.038344	0.042744373	0.023373443
Q8TBA6	GOLGA5	Golgin subfamily A member 5	3.828	1.93659083	0.042682	0.044062708	0.013417678
Q13322	GRB10	Growth factor receptor-bound protein 10	40.948	5.355721084	0.037881	0.042744373	0.058483667
A4D1B5	GSAP	Gamma-secretase-activating protein	6.293	2.653747943	0.031187	0.042744373	0.003264078
Q5T0I0	GSN	Gelsolin (Fragment)	6.248	2.643394453	0.032185	0.042744373	0.041406824
Q92522	H1-10	Histone H1.10	2.443	1.288653864	0.010008	0.136132152	0.226268486
P10412	H1-4	Histone H1.3	2.415	1.272023189	0.043274	0.044249643	0.111005064
P16401	H1-5	Histone H1.5	2.061	1.043344505	0.028405	0.042744373	0.112833286
Q5VWC8	HACD4	Very-long-chain (3R)-3-hydroxyacyl-CoA dehydratase 4	0.487	-1.038006323	0.001941	0.008169905	0.003102928
Q9NQE9	HINT3	Adenosine 5'-monophosphoramidase HINT3	25.493	4.672029254	0.037353	0.042744373	0.05646961
P09429	HMGB1	High mobility group protein B1	2.09	1.063502942	0.024365	0.042744373	0.008109876
Q9Y6F6	IRAG1	Inositol 1,4,5-triphosphate receptor associated 1	0.478	-1.064917477	0.004324	0.022167599	0.0167845
Q8WZA9	IRGQ	Immunity-related GTPase family Q protein	5.606	2.486971744	0.040186	0.042744373	0.067979098
O00522	KRIT1	Krev interaction trapped protein 1	5.141	2.362049012	0.008918	0.02337227	0.0101431
O00754	MAN2B1	Lysosomal alpha-mannosidase	27.657	4.789572768	0.019943	0.037734395	0.009922928
P55145	MANF	Mesencephalic astrocyte-derived neurotrophic factor	2.2	1.137503524	0.026014	0.042744373	0.036935389
O43318	MAP3K7	Mitogen-activated protein kinase kinase kinase 7	5.552	2.473007568	0.037689	0.042744373	0.034039738
P45984	MAPK9	Mitogen-activated protein kinase 9	5.144	2.362890643	0.008842	0.02337227	0.011740162
Q6ZWT7	MBOAT2	Lysophospholipid acyltransferase 2	4.142	2.050327554	0.038247	0.042744373	0.010510579
Q6DN14	MCTP1	Multiple C2 and transmembrane domain-containing protein 1	6.714	2.747172537	0.018355	0.036029711	0.019246801
Q8N3F8	MICALL1	MICAL-like protein 1	4.013	2.004681156	0.043064	0.044062708	0.05488591
Q96BX8	MOB3A	MOB kinase activator 3A	2.738	1.453122447	0.01148	0.042744373	0.02507774
Q8NDA8	MROH1	Maestro heat-like repeat-containing protein family member 1	8.732	3.12631213	0.00627	0.022026217	0.002085082
Q9BYD6	MRPL1	39S ribosomal protein L1, mitochondrial	3.875	1.95419631	0.042757	0.044062708	0.015106961
P49406	MRPL19	39S ribosomal protein L19, mitochondrial	3.172	1.665392771	0.047696	0.047346784	0.009634731
P00846	MT-ATP6	ATP synthase subunit a	0.485	-1.043943348	0.000098	0.022026217	0.013748808
P00403	MT-CO2	Cytochrome c oxidase subunit 2	0.473	-1.080087911	0.001134	0.008169905	0.003169091
P42898	MTHFR	Methylenetetrahydrofolate reductase (NADPH)	7.2	2.847996907	0.014341	0.028415625	0.005696085
P03891	MT-ND2	NADH-ubiquinone oxidoreductase chain 2	5.911	2.563402221	0.037457	0.042744373	0.068890298
P05976	MYL1	Myosin light chain 1/3, skeletal muscle isoform	0.439	-1.187707155	0.001275	0.042744373	0.074798488
O43920	NDUFS5	NADH dehydrogenase [ubiquinone] iron-sulfur protein 5	0.468	-1.095419565	0.000808	0.007500224	0.000732263
Q9NX40	OCIAD1	OCIA domain-containing protein 1	0.496	-1.011587974	0.003498	0.008169905	0.00091365
P20774	OGN	Mimecan	2.295	1.198494154	0.000389	0.007500224	0.002594607
Q969R2	OSBP2	Oxysterol-binding protein 2	25.989	4.699829218	0.039293	0.042744373	0.058894328
Q9BXB4	OSBPL11	Oxysterol-binding protein-related protein 11	4.39	2.13422094	0.022587	0.041240854	0.018731339
Q9NRP0	OSTC	Oligosaccharyltransferase complex subunit OSTC	0.481	-1.055891201	0.000521	0.008169905	0.002784679
Q9ULC6	PADI1	Protein-arginine deiminase type-1	31.029	4.955545297	0.037219	0.042744373	0.018262347
Q9NVE7	PANK4	4'-phosphopantetheine phosphatase	3.828	1.93659083	0.033944	0.042744373	0.069857169
Q8NEN9	PDZD8	PDZ domain-containing protein 8	34.875	5.124121312	0.018254	0.036029711	0.030582148
Q96S96	PEBP4	Phosphatidylethanolamine-binding protein 4	4.417	2.143066832	0.028753	0.042744373	0.020331597
O60568	PLOD3	Multifunctional procollagen lysine hydroxylase and glycosyltransferase LH3	4.192	2.067638717	0.023893	0.041821257	0.022962347
Q8TCS8	PNPT1	Polyribonucleotide nucleotidyltransferase 1, mitochondrial	21.312	4.413594082	0.039266	0.042744373	0.004822535
F2Z3J9	PTGR1	Prostaglandin reductase 1	3.768	1.913798965	0.050405	0.048871891	0.080495147
P23219	PTGS1	Prostaglandin G/H synthase 1	0.484	-1.046921047	0.000297	0.007500224	0.001339085
Q9Y3B8	REXO2	Oligoribonuclease, mitochondrial	21.288	4.41196851	0.038564	0.042744373	0.024262641
Q6NUQ1	RINT1	RAD50-interacting protein 1	6.595	2.721372659	0.011616	0.036029711	0.065615259
O14730	RIOK3	Serine/threonine-protein kinase RIO3	37.457	5.227163451	0.037514	0.042744373	0.056713515
Q96DM3	RMC1	Regulator of MON1-CCZ1 complex	124.761	6.963023211	0.000499	0.008169905	0.002064678
Q96DB5	RMDN1	Regulator of microtubule dynamics protein 1	7.949	2.990773378	0.006588	0.022167599	0.011055611
P07998	RNASE1	Ribonuclease pancreatic	2.006	1.004321606	0.000137	0.006976529	0.000615237
P34096	RNASE4	Ribonuclease 4	2.489	1.31556623	2.46E-06	0.000287099	1.95627E-05
P36955	SERPINF1	Pigment epithelium-derived factor	2.198	1.136191386	0.024133	0.041821257	0.02644979
Q9BRV8	SIKE1	Suppressor of IKBKE 1	15.693	3.972049271	0.041308	0.043583905	0.004794252
Q05940	SLC18A2	Synaptic vesicular amine transporter	5.822	2.541514839	0.017232	0.034113326	0.002594415
Q3KQZ1	SLC25A35	Solute carrier family 25 member 35	5.297	2.405175509	0.008017	0.022984919	0.010731801
Q9H4I9	SMDT1	Essential MCU regulator, mitochondrial	0.499	-1.002888279	0.002036	0.041821257	0.055954027
Q9Y343	SNX24	Sorting nexin-24	3.118	1.640620928	0.036852	0.042744373	0.036347463
Q9Y6A9	SPCS1	Signal peptidase complex subunit 1	0.468	-1.095419565	0.003831	0.041821257	0.039213253
P42226	STAT6	Signal transducer and activator of transcription 6	7.51	2.908812908	0.004751	0.019182115	0.004576747
Q9P2R7	SUCLA2	Succinate--CoA ligase [ADP-forming] subunit beta, mitochondrial	2.03	1.021479727	0.000229	0.007500224	0.001844533
O15533	TAPBP	Tapasin	0.471	-1.086201035	0.002602	0.008701906	0.013962727
Q92609	TBC1D5	TBC1 domain family member 5	14.92	3.89917563	0.047887	0.047346784	0.006017807
Q9Y4P3	TBL2	Transducin beta-like protein 2	5.585	2.481557281	0.038061	0.042744373	0.097049943

Q9NUJ3	TCP11L1	T-complex protein 11-like protein 1	82.082	6.358993979	0.008019	0.022984919	0.0064203
Q9Y6I9	TEX264	Testis-expressed protein 264	0.5	-1	0.000935	0.041821257	0.048719771
P35443	THBS4	Thrombospondin-4	2.258	1.175045486	0.000295	0.007500224	0.000629895
Q9Y584	TIMM22	Mitochondrial import inner membrane translocase subunit Tim22	3.845	1.942983598	0.046868	0.0471203	0.056520903
O43615	TIMM44	Mitochondrial import inner membrane translocase subunit TIM44	22.175	4.470862199	0.039554	0.042744373	0.052197008
Q9BTV4	TMEM43	Transmembrane protein 43	0.482	-1.052894948	0.000945	0.008169905	0.002881581
O94886	TMEM63A	CSC1-like protein 1	0.481	-1.055891201	0.000961	0.013913767	0.005717343
Q9Y5R8	TRAPPC1	Trafficking protein particle complex subunit 1	31.404	4.972876425	0.038149	0.042744373	0.058114194
P48553	TRAPPC10	Trafficking protein particle complex subunit 10	8.715	3.123500664	0.00629	0.022026217	0.002099357
Q86SZ2	TRAPPC6B	Trafficking protein particle complex subunit 6B	37.123	5.214241398	0.00808	0.022984919	0.002916548
Q96Q05	TRAPPC9	Trafficking protein particle complex subunit 9	6.304	2.656267535	0.017179	0.036029711	0.016233215
Q9NNW7	TXNRD2	Thioredoxin reductase 2, mitochondrial	3.103	1.633663697	0.038422	0.042744373	0.002454374
Q9BZI7	UPF3B	Regulator of nonsense transcripts 3B	21.718	4.440819347	0.034485	0.042744373	0.067885803
Q641Q2	WASHC2A	WASH complex subunit 2A	50.842	5.667948878	0.001367	0.010658114	0.004247359
Q9Y4P8	WIPI2	WD repeat domain phosphoinositide-interacting protein 2	55.647	5.798232007	0.037288	0.042744373	0.008211974
Q9Y2Z4	YARS2	Tyrosine--tRNA ligase, mitochondrial	7.202	2.8483976	0.015185	0.033639099	0.002555639

The data are presented as fold change (FC), logFC, unadjusted p values, Benjamini-Hochberg-adjusted p values, and covariate-adjusted p values after clinical confounder adjustment. Differential protein abundance analysis was performed using the limma package with empirical Bayes moderation.
